# Influence of correlated antigen presentation on T-cell negative selection in the thymus

**DOI:** 10.1098/rsif.2018.0311

**Published:** 2018-11-07

**Authors:** Soumya Banerjee, S. Jonathan Chapman

**Affiliations:** Mathematical Institute, University of Oxford, Oxford, UK

**Keywords:** negative selection, correlated antigen presentation, correlation, multiscale models

## Abstract

The thymus is the primary organ for the generation of naive T cells, a key component of the immune system. Tolerance of T cells to self is achieved primarily in the thymic medulla, where immature T cells (thymocytes) sample self-peptides presented by medullary thymic epithelial cells (mTECs). A sufficiently strong interaction activates the thymocytes leading to negative selection. A key question of current interest is whether there is any structure in the manner in which mTECs present peptides: can any mTEC present any peptide at any time, or are there particular patterns of correlated peptide presentation? We investigate this question using a mathematical model of negative selection. We find that correlated patterns of peptide presentation may be advantageous in negatively selecting low-degeneracy thymocytes (that is, those thymocytes which respond to relatively few peptides). We also quantify the probability that an auto-reactive thymocyte exits the thymus before it encounters a cognate antigen. The results suggest that heterogeneity of gene co-expression in mTECs has an effect on the probability of escape of autoreactive thymocytes.

## 1. Introduction

The thymus is the primary organ for the generation of naive T cells, a key component of the immune system. T cells play a key role in the adaptive immune response, combating pathogens that have invaded host cells. Pathogen-derived proteins in infected host cells are processed into short peptides (p) which can then bind to major histocompatibility (MHC) proteins. The resulting peptide–MHC (pMHC) complexes are presented on the surface of the host cell, ready for interrogation by T cells [1].

T cells express a protein on their surface called the T-cell receptor (TCR). Each TCR has a highly variable region (the CDR3 region) which is responsible for antigen recognition. This region is generated randomly during the T-cell maturation process through stochastic gene rearrangement, with the result that each T cell expresses a distinct TCR. A given T cell is said to recognize a particular pMHC complex if its TCR binds sufficiently strongly to it to enable downstream signalling cascades inside the T cell that result in its activation and proliferation [1].

Host proteins are also processed into short peptides and may be presented as pMHC complexes. To prevent T cells attacking the host it is important to eliminate from the pool of naive T cells those which recognize self-antigens. This occurs in the thymus in a process called central tolerance.

To purge the pool of immature T cells (thymocytes) of cells with a reactivity to self-antigens, antigen presenting cells in the thymus (primarily medullary thymic epithelial cells, mTECs) provide a comprehensive ‘molecular library’ of self-antigens that, when recognized by developing, self-reactive T cells, will initiate their death. These cells promiscuously express the self-transcriptome at the single-cell level [2]. This deletion of potentially harmful T cells is known as thymic negative selection and prevents the formation of effector T cells able to initiate an injurious autoimmune response.

In a series of papers [3–6], Košmrlj *et al.* have developed a computational model for this process of negative selection. The interaction between a TCR and a pMHC complex is modelled as follows. The more conserved region of the TCR, which interacts with the MHC molecule directly, is not modelled explicitly; the interaction energy due to this component is assigned a value *E*_*c*_. In the simplest incarnation of the model, this value is a fixed parameter, while in more complex versions it may be drawn randomly from a given distribution of interaction energies. The highly variable region of the TCR, and the peptide bound to the MHC, are each modelled as a string of amino acids. Each site on the TCR is taken to interact with the corresponding site on the peptide, with an interaction energy depending on the two amino acids (*t* and *r*, say) given by the Miyazawa–Jernigan matrix *J*(*t*, *r*) [7,8]. The energy of interaction of the TCR–pMHC pair is then given by the sum of all these individual energies:1.1
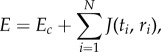
where *J*(*t*_*i*_, *r*_*i*_) is the contribution of the *i*th amino acid on the TCR (*t*_*i*_) and the peptide (*r*_*i*_), and there are *N* binding sites in total. Although the typical length of a peptide is nine amino acids, only positions 3–7 are taken to be available for binding to the TCR, with the remaining residues responsible for binding to the MHC groove or buried within the groove; thus *N* is chosen to be 5, consistent with experimental data [9] and prior modelling work [3,4].

Košmrlj *et al.* then perform a number of numerical experiments. They first randomly create a set of *M* peptides to represent the self-peptides presented in the thymus. They then randomly generate a set of TCRs and select them against these peptides. This process is repeated many times to generate statistical results. In this way, they are able to predict that TCRs which survive negative selection are enriched in weakly binding amino acids, and that the pathogens they recognize are enriched in strongly binding amino acids. Both of these effects increase strongly with the number of self-peptides the TCRs are selected against. In their simulations, each TCR interacts with all antigens in the thymus. They do not consider the question of a TCR randomly evading negative selection by exiting the thymus before it had a chance to interact with a cognate antigen.

With the advent of experimental techniques generating single-cell gene expression data, there has been much recent interest in determining the manner in which mTECs present the self-transcriptome [2]. Individual mature mTECs show patterns of gene co-expression [10,11]. Is each mature mTEC capable of presenting any self-antigen, or are there a number of different classes of mTEC which divide up the space of self-antigens between them? Is promiscuous gene expression by mTECs stochastic, either spatially or temporally?

**Figure 1.**
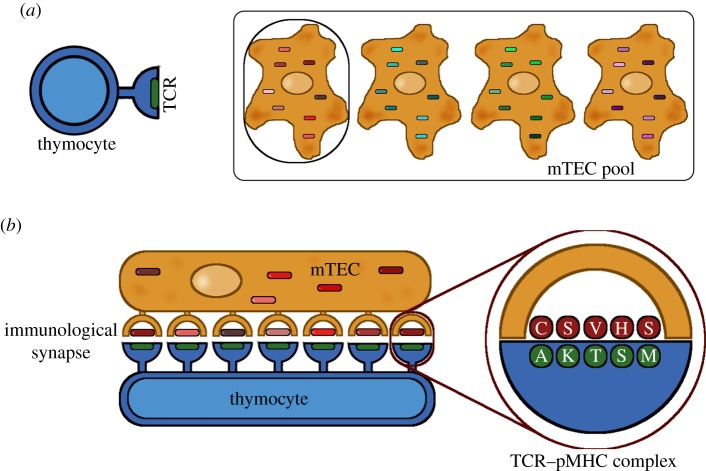
Schematic illustration of the model. In (*a*), the thymocyte randomly chooses an mTEC from the pool for its next interaction. Each mTEC is endowed with a gene expression profile, which determines the peptides it can present. There are *K* different profiles, giving *K* different classes of mTEC (four are illustrated). The interaction is illustrated in (*b*). The mTEC decides randomly which peptides to present. There are *s* TCR–pMHC complexes in the immunological synapse (seven are illustrated). The energy of interaction of each complex is determined by summing the pairwise interaction energies of amino acids in the peptide and the corresponding amino acids in the CDR3 region of the TCR. The thymocyte is negatively selected if enough complexes exceed a critical interaction energy. If the thymocyte is not negatively selected, it proceeds to the next interaction, randomly choosing another mTEC.

The model of Košmrlj *et al.* focuses on individual TCR–pMHC interactions, rather than T cell–mTEC interactions. In reality, a T cell will undergo a sequence of interactions with mTECs as it progresses through the thymus [12–14]. For each interaction, an immunological synapse will form comprising a large number of TCR–pMHC interactions, of the order of 2000 [15] (figure 1). The response of the T cell will depend on the interaction energies of each of these interactions, {*E*_*j*_: *j* = 1, …, 2000}, say. The model of Košmrlj *et al.* is equivalent to assuming that the T cell will be negatively selected if the energy of any one interaction exceeds a threshold, that is, 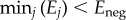 (note that interaction energies are negative). Other authors have assumed that a T cell responds to the triggering rate averaged over all its TCRs, where the triggering rate of a given TCR–pMHC complex is a function of its interaction energy [16].

Our focus in this paper is to extend the model of Košmrlj *et al.* to incorporate the fact that an immunological synapse comprises many TCR–pMHC interactions, and that these may not be all independent. We focus in particular on two effects: (i) the peptides presented may not be independent, since they are presented by mTECs, which individually have patterns of gene expression [2,10,11] and (ii) the response of the T cell may depend on the complete set of interaction energies, rather than on any one exceeding a threshold. Our goal is to determine whether differential gene expression of mTECs has a discernible effect on the probability of escape of autoreactive T cells.

Although we will populate our synthetic thymus with randomly generated peptides in the same way as Košmrlj *et al.*, the model is designed in such a way that it is capable of incorporating single-cell genomic or proteomic data when they become available.

## 2. Material and methods

### 2.1. Summary of methods

We have created a computational modelling framework to simulate the presentation of self-peptides on the surface of mTECs and interaction of these peptides with the CDR3 region of TCRs on thymocytes. The overview of this framework is as follows. We first generate the set of all self-peptides. We then create a set of virtual mTECs each of which is able to present (some or all) peptides from this set. Immature T cells (thymocytes) are generated, each having a random amino acid sequence for the CDR3 region of their TCRs. Each thymocyte chooses a sequence of mTECs to interact with, and during each interaction each mTEC chooses a set of peptides to present. If the interaction is too strong, the thymocyte is deleted (negative selection). The model is illustrated schematically in figure 1.

### 2.2. Generating a set of self-peptides

We randomly generated a set of *m* peptides (we consider the cases *m* = 10 000 and *m* = 100 000; see section Model parameters). Each peptide comprises a string of *N* amino acids (in our examples *N* = 5 [4]). Each amino acid is chosen randomly with a probability proportional to the frequency of amino acids in the mouse proteome [3]. This set of peptides is considered to be the set of all self-peptides.

We then suppose that there are *K* different types of mTEC in the thymus, and that the set of *m* self-peptides is divided up between them without overlap. In reality, we would expect that even if there were a number of different classes of mTEC each with its own gene expression profile, there would be some overlap in the peptides they could present. However, in the absence of any specific experimental evidence, we choose a model without overlap as a representative extreme case of mTEC specialization. We investigate the cases *K* = 1 (corresponding to no specialization, so that all mTECs are able to present all peptides), and *K* = 10, 100 and 1000. We note that experiments suggest that the thymus might contain autonomous tolerogenic units comprising approximately 200 distinct mTECs [17], though of course, the peptide presentation capabilities of the mTECs in each unit are not known.

Each *in silico* mTEC thus created has a set of peptides associated with it from which it chooses peptides to present to thymocytes.

### 2.3. Stochastic simulator

We simulate the passage of a thymocyte through the thymic medulla and its resulting interaction with the pool of *in silico* mTECs. The thymocyte is given a TCR sequence comprising *N* randomly chosen amino acids (with each amino acid chosen with a probability proportional to its frequency in the mouse proteome [3]). The simulator then randomly picks a sequence of mTECs from the *in silico* pool to interact with the thymocyte.

For each thymocyte–mTEC interaction, we suppose that there are *s* TCR–pMHC complexes in the immunological synapse [15,16]; we vary *s* in the range 100–2000. For each MHC in the synapse, we randomly choose a peptide for it to present, with a probability distribution proportional to the relative peptide abundances in this particular mTEC.

We follow [3–6] and evaluate the interaction energy of each TCR–pMHC complex by summing over the interaction energies of the exposed amino acids, using the Miyazawa–Jernigan matrix of interaction energies [7,8] as in equation (1.1). The fate of the thymocyte is then determined by the collection of energies {*E*_*j*_: *j* = 1, …, *s*}, say. In the simplest case, corresponding to that used in [3–6], if any one energy exceeds a threshold *E*_neg_ the thymocyte is deemed to have been negatively selected and is deleted. We investigate the effect of more complex selection rules by considering also the case in which at least *p* energies must exceed a threshold; we vary *p* in the range 1–3.

The model is illustrated schematically in figure 1, and in pseudo-code in algorithm 1.


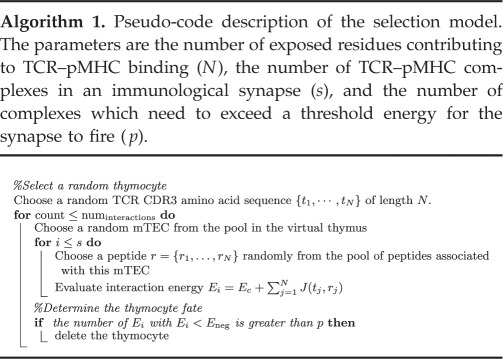


### 2.4. Model parameters

We summarize here the key model parameters, and our estimates for them.

*m*: the total number of peptides. We vary the total number of peptides from a value of 10 000 (estimated from gene expression data using older sequencing techniques suggesting that 2000 genes are expressed in the medulla [18]) to 100 000 (estimated from a recent and more sensitive single-cell gene expression study suggesting that approximately 20 000 genes are expressed in the medulla [2]).

*s*: the number of TCR–pMHC complexes engaged in the immunological synapse. This is varied between 100 and 2000 based on experimental estimates [15].

*N*: the length of the region of the peptide that is exposed and available for binding. The typical length of peptides presented by mTECs (bound to MHC-I) is nine amino acids. We assume that the third through the seventh amino acids are available for binding to the TCR CDR3 region, consistent with experimental data [9] and prior modelling work [3]. Hence in our models we set *N* = 5.

*K*: number of distinct classes of mTECs in the thymus (that is, the number of distinct gene-expression profiles). Experiments suggest that approximately 200–500 distinct mTECs exist in autonomous tolerogenic units [17,19], which would suggest an upper bound for *K* in this region. The lower bound is simply *K* = 1, corresponding to all mTECs being identical and each able to express any gene. We vary *K* over the range 1–1000.

num_interactions_: the number of interactions between a given thymocyte and a series of mTECs. We can make some estimate of the number of interactions that are biologically feasible. Thymocytes reside in the medulla for approximately 4 days [17]. The immunological synapse lasts for approximately 30 min [20]. This sets an upper bound on the number of interactions a thymocyte can have with mTECs to approximately 200. However, the actual number of feasible interactions is likely to be far fewer after accounting for thymocyte migration time. We vary the number of interactions from 1 to 100.

*p*: the minimum number of peptides presented by an mTEC that must simultaneously interact sufficiently strongly with the TCR for the thymocyte to be negatively selected. We present results for *p* in the range 1–3.

*E*_neg_: the negative selection energy threshold. This is chosen so that a biologically realistic proportion of all thymocytes entering the medulla survive negative selection.

The fraction of all thymocytes entering the medulla (after VDJ recombination and after positive selection) that survive negative selection is2.1
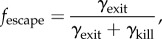
where *γ*_exit_ is the rate at which surviving thymocytes exit the medulla and *γ*_kill_ is the rate at which thymocytes are deleted (by negative selection) in the medulla. Experimental data in [21] suggest that *γ*_exit_ = 2.9 × 10^6^ per day and *γ*_kill_ = 4.8 × 10^6^ per day, which gives *f*_escape_ = 38%. In [22], *f*_escape_ is estimated directly at 5%.

Matching this range of values of *f*_escape_ to the probability that a thymocyte survives negative selection in our stochastic simulation, gives a negative selection energy threshold (*E*_neg_) between −20.0 *k*_b_*T* and −24.0 *k*_b_*T*. We use *E*_neg_ = − 21.0 *k*_b_*T* in our simulations.

## 3. Results

### 3.1. The degeneracy of the TCR–pMHC interaction

**Figure 2.**
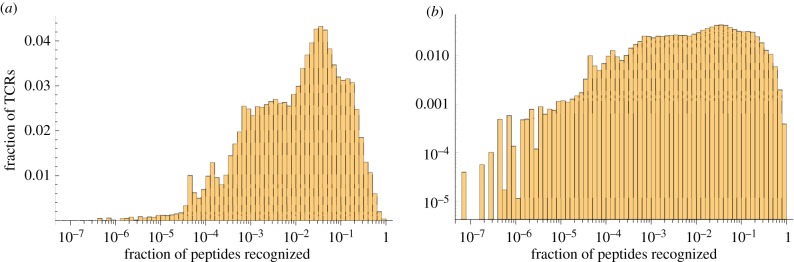
Distribution of the proportion of peptides recognized by each of 200 000 randomly generated TCR sequences shown on (*a*) log–linear and (*b*) log–log scales. Model parameters used are *E*_neg_ = − 21.0 *k*_b_*T* and *N* = 5. The proportion of TCRs not recognizing any peptides was approximately 12%.

The interaction between a TCR and pMHC is degenerate: each TCR will recognize a number of different peptides bound to MHC class I. To try and quantify this we generated 200 000 random TCR sequences and calculated for each one the probability of it recognizing a randomly generated peptide. We show in figure 2 the resulting distribution of the fraction of peptides recognized by each TCR. We see that (for these parameter values) the majority of TCRs are highly degenerate, recognizing over 1% of all self-peptides, while there are a few low-degeneracy TCRs that recognize fewer than one in 1000 peptides, corresponding to tens or hundreds of self-peptides.

The distribution of degeneracy illustrated in figure 2 is all the information we need from the detailed model of an individual TCR–pMHC interaction. For the paired amino acid model we are using, this distribution depends on the two parameters *E*_neg_ and *N*. Let us briefly examine the effect of varying these parameters.

We show in electronic supplementary material, figure S1, the degeneracy distribution when *N* = 5 for *E*_neg_ = − 20.0 *k*_b_*T*, −21.0 *k*_b_*T*, −22.0 *k*_b_*T*, −23.0 *k*_b_*T*, and −24.0 *k*_b_*T*. We see that lower thresholds correspond to a small shift in the distribution so that there are fewer high-degeneracy TCRs and more low-degeneracy TCRs. In addition, there is an increase in the number of TCRs which do not recognize any peptide (so would not be positively selected). Since lower degeneracy TCRs are harder to detect, this change in distribution will have a knock-on effect on the overall probability that a randomly chosen TCR escapes negative selection.

We show in electronic supplementary material, figure S2, the degeneracy distribution when *N* = 9 for *E*_neg_ = − 32.0 *k*_b_*T*, −35.0 *k*_b_*T*, −38.0 *k*_b_*T*, and −41.0 *k*_b_*T* (since there are more amino acids contributing to binding, we need to scale the negative energy selection threshold accordingly). We see the same trend as before, that lower thresholds correspond to a shift in the distribution so that there are fewer high-degeneracy TCRs and more low-degeneracy TCRs. Comparing electronic supplementary material, figures S2 to S1, we see that the distribution is ‘smoother’: we are closer to the 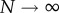 limit in which the distributions can be approximated using statistical mechanics [6]. There is also a longer tail: with nine amino acids there are many more possible peptides and so it is possible to have TCRs which recognize fewer that 10^−9^ peptides (but greater than zero).

### 3.2. A first look at the effect of correlation

We discuss here the impact of our modification to the model of [3–6], namely that the distribution functions for the peptides presented on a given mTEC may not be independent. To illustrate the effect of correlation, suppose first that there is only one type of mTEC, so that each mTEC can present any peptide (*K* = 1). Suppose also that *p* = 1, so that if any TCR–pMHC complex in the immunological synapse exceeds the threshold energy then the thymocyte will be negatively selected. If there are *m* self-peptides available for presentation, and *s* TCR–pMHC complexes, then the probability that a given TCR sequence of degeneracy *d* (i.e. recognizing *d* self-peptides) is not deleted in the thymus after *n* interactions is given by3.1
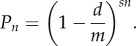
Note that this formula depends only on the product *sn*: interacting with 1000 peptides presented on 1 mTEC is equivalent to interacting with 1000 mTECs each presenting 1 peptide; only the total number of peptides presented matters.

Now suppose that there are *K* > 1 distinct classes of mTEC, each capable of presenting *m*/*K* different peptides. We denote the degeneracy of the TCR sequence with respect to the *i*th mTEC by *d*_*i*_, that is, we suppose that out of the *m*/*K* peptides the *i*th mTEC can present, the TCR recognizes *d*_*i*_ peptides, where 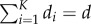. Given *d* (via figure 2) and a model for the partition of peptides between mTECs (we assume a random partition) the distribution function for the vector (*d*_1_, …, *d*_*K*_) can be calculated. The probability of escape after *n* interactions is now given by3.2
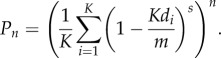
If *d*_*i*_ = *d*/*K*, so that the degeneracy is the same for each mTEC, then (3.2) is equal to (3.1). But there is no reason to suppose that this is the case.

On the other hand, if the chance of being negatively selected in one interaction is small, so that *Kd*_*i*_*s*/*m* ≪ 1, we find
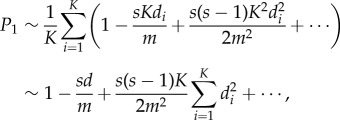
and the dependency on *K* comes only in the third term, so that *d*_*i*_ being non-uniform across mTECs has a relatively small effect on the probability of negative selection.

The formulae above are modified slightly if at least *p* TCR–pMHC complexes must simultaneously exceed the negative selection energy threshold for the thymocyte to be negatively selected, with *p* > 1. The probability that a TCR escapes after *n* interactions is now3.3
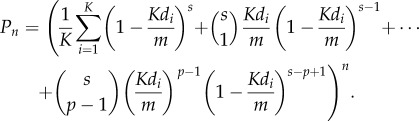
For small *Kd*_*i*_*s*/*m*, equation (3.3) gives
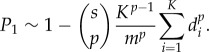
Note that in this case the non-uniformity of *d*_*i*_ across mTECs affects the probability of negative selection much more strongly, and the effect increases with increasing *p*. Thus, the effect of correlations, and the question of whether there is specialization among mTECs, is much more important when multiple pMHCs need to be recognized simultaneously in order for a thymocyte to be negatively selected.

We will now determine more quantitatively how the probability of escape depends on the parameters in the model. We first examine the probability of escape for a TCR of a given degeneracy *d*, under the assumption that the available peptides are randomly partitioned among the *K* mTEC classes. This analysis is independent of the particular detailed (amino acid based) model for activation of a TCR–pMHC complex. We then combine this with the distribution of degeneracy illustrated in figure 2 to determine the probability of escape of a random TCR over multiple interactions.

### 3.3. The probability of escape in a single interaction

**Figure 3.**
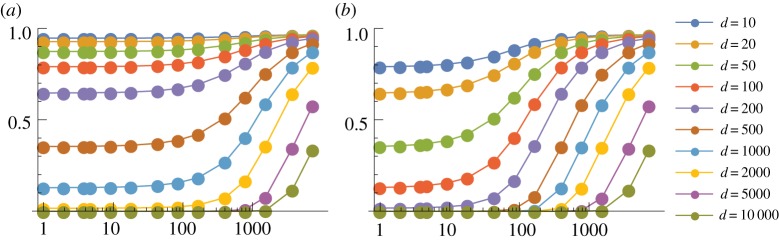
Mean probability of escape in a single interaction, *P*_1_, as a function of the number of mTEC classes, *K*, for various TCR degeneracies, *d*. Other model parameters are *m* = 100 000, *p* = 1. A wider range of values of *s* are illustrated in electronic supplementary material, figure S3. (*a*) *s* = 200 and (*b*) *s* = 2000.

**Figure 4.**
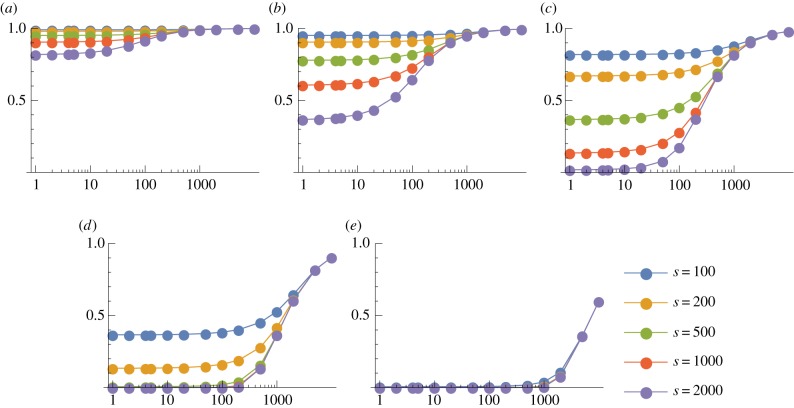
Mean probability of escape in a single interaction, *P*_1_, as a function of the number of mTEC classes, *K*, for various numbers of TCR–pMHC complexes in the immunological synapse, *s*. Other model parameters are *m* = 100 000, *p* = 1. A wider range of values of *d* are illustrated in electronic supplementary material, figure S4. (*a*) *d* = 10, (*b*) *d* = 50, (*c*) *d* = 200, (*d*) *d* = 1000 and (*e*) *d* = 5000.

We first consider the case *p* = 1, so that a thymocyte is negatively selected if any one of its TCRs interact strongly with the corresponding pMHC complex. We show in figures 3 and 4 the probability of escape in a single thymocyte–mTEC interaction (*P*_1_) as a function of the number of mTEC classes (*K*) for various numbers of TCR–pMHC complexes in the immunological synapse (*s*) and a range of TCR degeneracies (*d*). Results are shown here for *m* = 100 000 self-peptides; corresponding plots for *m* = 10 000 are given in electronic supplementary material, figures S5 and S6. Some obvious trends are observed in the data: the more degenerate a TCR, and the more TCR–pMHC complexes in the immunological synapse the less likely the thymocyte is to escape. We also see that TCRs with a degeneracy over 5000 (i.e. those which react with over 5% of all peptides) are very likely to be negatively selected by just one thymocyte–mTEC interaction. For large enough *s*, there is also a threshold effect: the probability of escape transitions from zero to one around the value *K* ≈ *d*. We explain this behaviour in the following section (see equation (3.4)). More generally, the dependence on the number of mTEC classes *K* is monotonic: the value of *K* which leads to the lowest probability of escape of an autoreactive thymocyte is *K* = 1. Thus, if we imagine the different ways that self-peptides could be divided among mTECs, the optimal strategy when *p* = 1 is to have any mTEC capable of presenting any peptide.

**Figure 5.**
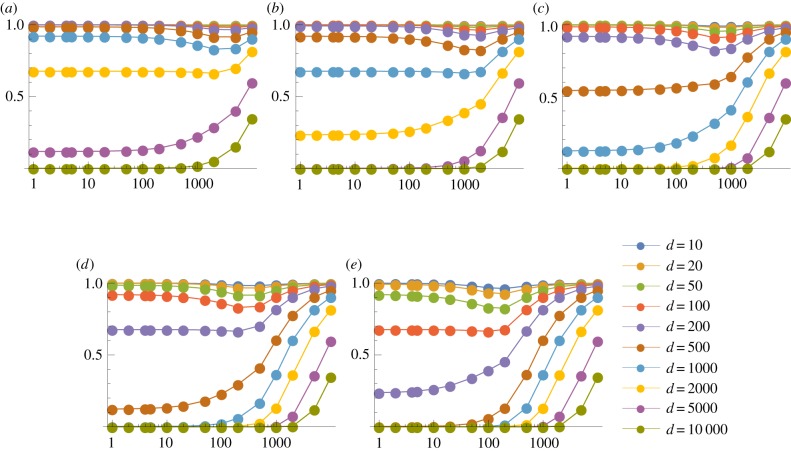
Mean probability of escape in a single interaction, *P*_1_, as a function of the number of mTEC classes, *K*, for various TCR degeneracies, *d*. Other model parameters are *m* = 100 000, *p* = 3. (*a*) *s* = 100, (*b*) *s* = 200, (*c*) *s* = 500, (*d*) *s* = 1000 and (*e*) *s* = 2000.

**Figure 6.**
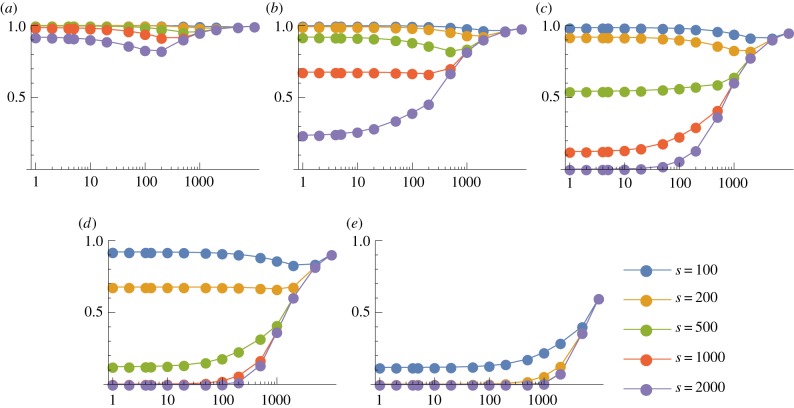
Mean probability of escape in a single interaction, *P*_1_, as a function of the number of mTEC classes, *K*, for various numbers of TCR–pMHC complexes in the immunological synapse, *s*. Other model parameters are *m* = 100 000, *p* = 3. A wider range of values of *d* is illustrated in electronic supplementary material, figure S11. (*a*) *d* = 50, (*b*) *d* = 200, (*c*) *d* = 500, (*d*) *d* = 1000 and (*e*) *d* = 5000.

We now consider the case *p* = 3, so that a thymocyte is negatively selected if and only if at least three of its TCRs interact strongly with the corresponding pMHC complexes. (The corresponding results for *p* = 2 are given in electronic supplementary material, figures S7–S10.) We show in figures 5 and 6 the probability of escape in a single thymocyte–mTEC interaction as a function of the number of mTEC classes for various *s* and *d* when there are *m* = 100 000 self-peptides; corresponding plots for *m* = 10 000 are given in electronic supplementary material, figures S12 and S13. While it is still true that the larger *d* and *s* the less likely the thymocyte is to escape (as we would expect), the behaviour as the number of mTEC classes *K* varies is more interesting. For large *d* and *s*, the behaviour is still monotonic in *K*, so that the optimal strategy is still *K* = 1. But for low degeneracies or low numbers of TCR–pMHC complexes in the immunological synapse, there is an optimal value of *K* which minimizes the probability of escape. We will find in our analysis below that, roughly speaking, for degeneracies *d* ⪆ *m*/*s* to minimize the probability of escape it is best to choose *K* = 1 so that all mTECs can present all peptides, while for degeneracies *d* ⪅ *m*/*s* it is best to divide the mTECs into *K* ≈ *m*/*s* classes, each of which can present around *s* peptides.

#### 3.3.1. Mathematical analysis

Providing *s* is not too small, *P*_1_ given by (3.2) is dominated by the probability that one of the *d*_*i*_'s is small. For *s* ≫ *m*/*K* (in practice, for *s* ≥ 2*m*/*K*), *P*_1_ is completely determined by the probability that one of the *d*_*i*_'s is zero: in this limit, the complete set of peptides within a cell is almost certain to be expressed, so the only way to avoid negative selection is to choose a cell in which there is no matching peptide.

Choosing *m*/*K* peptides, each with a probability *d*/*m* of matching, gives3.4
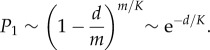
The collapse of the data onto this simple expression is illustrated in electronic supplementary material, figure S14.

When *s* is not so large as this, we need to take account of the fact that if an mTEC has small (rather than zero) degeneracy a matching peptide may not be presented.

**Figure 7.**
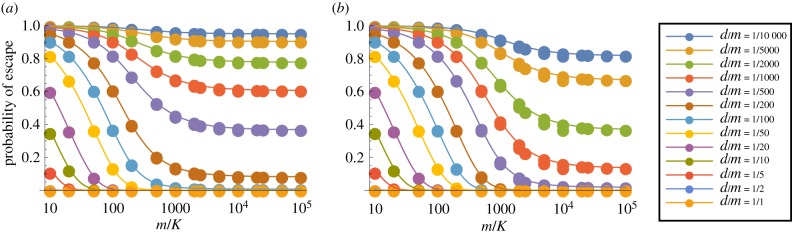
The mean probability of escape as a function of *m*/*K*. The curves are given by equation (3.5). The points are calculated values, using both *m* = 10^4^ and 10^5^. For (*a*) *s* = 500, these points lie on top of one another. For (*b*) *s* = 2000, the difference between the two sets of points is just visible near *m*/*K* = 10^4^. Here *p* = 1.

In this case,3.5
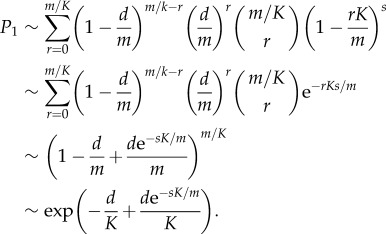
We see that the four parameters *d*, *K*, *s* and *m* only appear in the combinations *d*/*K* and *sK*/*m*. This allows us to collapse the results shown in electronic supplementary material, figures S6 and figure 4 to a single plot. Sample plots are shown in figure 7, in which the data are compared to equation (3.5).

**Figure 8.**
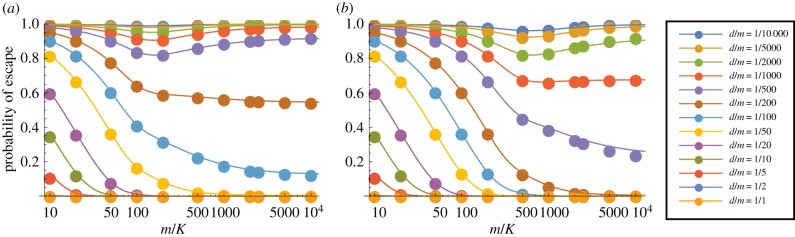
The mean probability of escape as a function of *m*/*K*. The curves are given by equation (3.7). The points are calculated values, using both *m* = 10^4^ and 10^5^. These points lie on top of one another. Here *p* = 3. (*a*) *s* = 500 and (*b*) *s* = 2000.

The corresponding results for *p* = 2 and *p* = 3 are derived in the electronic supplementary material, appendix S1, and are3.6
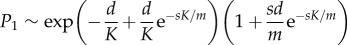
and3.7
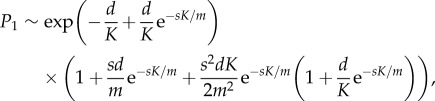
respectively. Again these depend only on the combinations *d*/*K* and *sK*/*m*. The data are compared to equation (3.7) in figure 8 (and equation (3.6) in electronic supplementary material, figure S15).

**Figure 9.**
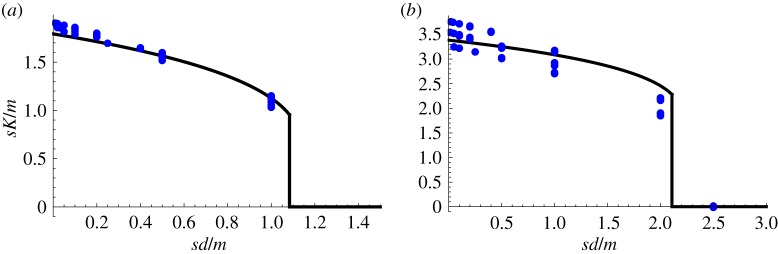
The optimal value of *sK*/*m* as a function of *sd*/*m*, for (*a*) *p* = 2 and (*b*) *p* = 3. The points are calculated values for *m* = 10^4^ (shown in electronic supplementary material, figures S12 and S13) and *m* = 10^5^ (shown in figures 5 and 6). The curves are given by equations (3.8) and (3.9), respectively. Roughly, we see that for degeneracies *d* ⪆ *m*/*s* it is best to choose *K* = 1 so that all mTECs can present all peptides. For degeneracies *d* ⪅ *m*/*s*, it is best to divide the mTECs into *K* ≈ *m*/*s* classes, each of which can present around *s* peptides.

We can use these formulae to find analytically the optimal value of *K*. When *p* = 1, if we denote *x* = *sK*/*m*, then *P*_1_ is minimized when
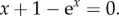
This gives *x* = 0, so that *K* should be made as small as possible. When *p* = 2, if we denote also *y* = *sd*/*m*, then *P*_1_ is minimized when3.8
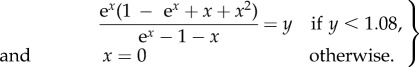
When *p* = 3, *P*_1_ is minimized when3.9
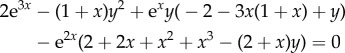
if *y* < 2.11 and *x* = 0 otherwise. These optimal values are illustrated in figure 9. As claimed, we see that, roughly speaking, for degeneracies *d* ⪆ *m*/*s* it is best to choose *K* = 1 so that all mTECs can present all peptides. For degeneracies *d* ⪅ *m*/*s*, it is best to divide the mTECs into *K* ≈ *m*/*s* classes, each of which can present around *s* peptides. The reason for this is as follows. For low degeneracies, the chance of finding two or more matching peptides in a random sample of *s* peptides from the whole pool becomes small. But if an mTEC has a limited repertoire of peptides then, if this set happens to contain one matching peptide, there is a much more significant chance that two copies of it will be presented.

While this result is intriguing, we do not claim that evolution would necessarily select this optimal configuration, as there are many other factors to take into account. Even within our model, with reference to figure 5*d*, for example, we see that choosing *K* = 200 rather than *K* = 1 reduces the probability of escape of thymocytes with degeneracy 100, but increases significantly the probability of escape of thymocytes with degeneracy 500. The overall efficiency of negative selection will depend on the degeneracy distribution of the incoming thymocytes (figure 2). We examine this in more detail in the following section. Moreover, the evolutionary pressure itself is not necessarily clear: low-degeneracy autoreactive TCRs may also be less dangerous than high-degeneracy TCRs in the periphery, for example.

### 3.4. Probability of autoreactivity

Having looked in detail at the probability of escape in a single interaction, we now consider a sequence of T-cell–mTEC interactions to determine the number of such interactions required for negative selection.

**Figure 10.**
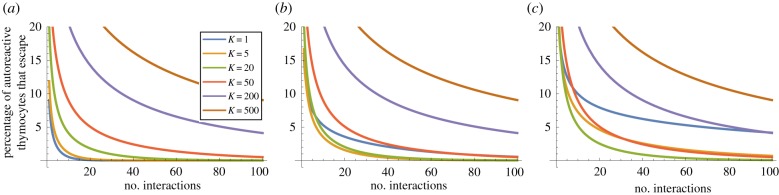
Percentage of all autoreactive thymocytes that escape as a function of the number of interactions with mTECs. Model parameters are *N* = 5, *m* = 10 000, *E*_neg_ = − 21.0*k*_b_*T*, *s* = 2000. (*a*) *p* = 1, (*b*) *p* = 2 and (*c*) *p* = 3.

**Figure 11.**
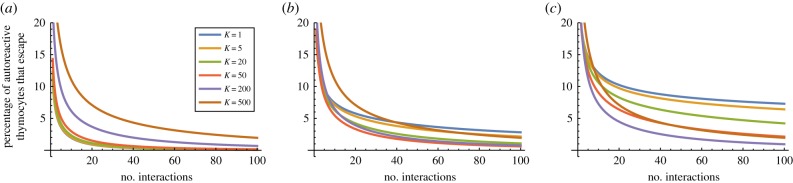
Percentage of all autoreactive thymocytes that escape as a function of the number of interactions with mTECs. Model parameters are *N* = 5, *m* = 100 000, *E*_neg_ = − 21.0*k*_b_*T*, *s* = 2000. (*a*) *p* = 1, (*b*) *p* = 2 and (*c*) *p* = 3.

For a given degeneracy *d*, if the probability of escape after one interaction is *P*_1_(*d*), then the probability of escape after *n* interactions is simply *P*_1_(*d*)^*n*^. Thus to find the probability of escape after *n* interactions, we need to average *P*_1_(*d*)^*n*^ over the distribution of degeneracy *d* (as illustrated in figure 2). The percentage of T cells surviving after each interaction is shown in figures 10 and 11 for a representative set of parameters (*E*_neg_ = − 21.0 *k*_b_*T*, *s* = 2000, and *m* = 10 000 and *m* = 100 000, respectively).

We observe that the majority of autoreactive T cells are negatively selected within just a few interactions, but that there are a few (low degeneracy) autoreactive T cells which take much longer to eliminate. As we might expect, the simple threshold model (*p* = 1) eliminates T cells more rapidly than the models requiring multiple matches (*p* > 1), since the criterion for negative selection is more readily satisfied. We also see that if the number of self-peptides is increased from *m* = 10 000 to *m* = 100 000, then negative selection takes longer, since the fraction of self explored in each interaction is smaller. If we suppose that for each mTEC–thymocyte interaction the immunological synapse lasts for 30 min [20], and assume it takes approximately 30 min for the synapse to dissociate and the thymocyte to migrate and find another mTEC to interact with, then the number of interactions corresponds exactly to the number of hours since the thymocyte entered the medulla. The horizontal axis in figures 10 and 11 then covers 4 days.

We also see confirmation that when *p* > 1 it can be beneficial to divide peptides among a number of mTEC classes rather than have all mTECs identical: figures 10*b*,*c* and 11*b*,*c* show that *K* = 1 is less efficient at eliminating autoreactive T cells than *K* = 5, *K* = 20, *K* = 50 and *K* = 200, respectively.

## 4. Conclusion and discussion

We have implemented a model of thymic selection in which decisions are made based on the interaction energies of multiple TCR–pMHC complexes in the immunological synapse, as indicated in the experimental results of [12–14]. The model computationally recapitulates the complex process of T-cell negative selection in the thymus through a series of interactions between thymocytes and mTECs presenting self-peptides.

The detailed energetic model of an individual TCR–pMHC interaction is used to calculate the distribution of degeneracy of a random TCR against all possible peptides, that is the probability distribution for the proportion of peptides which would activate a randomly generated TCR. This distribution is all that is needed to model multiple interactions, both in parallel (through multiple TCR–pMHC interactions on a given mTEC) and in series (through sequential interactions with different mTECs).

The typical length of peptides presented by mTECs (bound to MHC-I) is 9 amino acids. In this study, we assume that the third through the seventh amino acid are available for binding to the TCR CDR3 region, following prior modelling work [3]. Recent experimental evidence suggests that the number of contacts between the TCR–pMHC complex is concentrated around a region consisting of approximately five to six amino acids [9]. To consider interactions with MHC-II complexes, which are relevant for many autoimmune diseases [23,24], we would have to include more amino acids in the binding region. We show the effect on the degeneracy distribution of including nine binding amino acids in electronic supplementary material, figure S2.

The model of the TCR–pMHC complex we adopted is (necessarily) a gross simplification: in reality, the three-dimensional structural properties of the TCR–pMHC complex are likely to be important [25–27], and may be poorly accounted for by simple pairwise amino acid interactions. More realistic models will require a great deal of data to parametrize, either experimental or from molecular dynamics simulations. Our analysis has identified that the key output of any such improved model is the degeneracy distribution of TCRs.

For the parameters considered, we found that 12% of TCR sequences did not recognize any peptide. Of the remainder, many TCR sequences have high degeneracy (half of all TCRs interact with more than 1.3% of peptides) but that there are a few low degeneracy TCRs (5% of TCRs interact with fewer than 1 in 10 000 peptides; figure 2). Our model indicates that many TCR sequences are negatively selected very quickly, within 10–15 interactions with mTECs in the medulla (figures 10 and 11), but that there are some (of low degeneracy) which take many more interactions with mTECs to find their cognate peptides and be deleted.

Mature mTECs co-express genes and show genomic clustering [10,11]. A key question of current interest is whether gene expression by mTECs is stochastic in time and/or space, and whether there is correlation between the genes expressed by different mTECs. To investigate the impact that such effects might have on negative selection, we investigated two alternative scenarios in our model. In the first, there was no specialization or correlation among mTECs: each mTEC could express any gene at any time so that its presented peptides were chosen randomly from all self-peptides. In the second, the space of all self-peptides was divided up among *K* different classes of mTEC, without overlap. For example, if there were 10 000 self-peptides and two classes of mTEC, we imagined that an mTEC from the first class could present peptides 1–5000, and mTEC from the second class could present peptides 5001–10 000. These classes do not necessarily correspond to different cell types: all mTECs may be the same but they may have a number of different possible gene expression profiles and switch between these (perhaps randomly) over time.

The impact of such correlation in the gene expression profiles of mTECs depends on the number of TCRs which need to be triggered in the immunological synapse for negative selection to occur. If only one TCR needs to be triggered, then the most efficient strategy is to have no correlation, so that all mTECs are capable of expressing all self-peptides at any time (*K* = 1). However, if more than one TCR needs to be triggered, then, depending on the parameters, it can become more efficient to correlate the self-peptides which may be co-expressed (*K* > 1). Specifically, we find that if there are *m* self-peptides and *s* TCR–pMHC complexes in the immunological synapse, then for TCRs of sufficiently high degeneracy (*d* ⪆ *m*/*s*) it is best to choose *K* = 1 so that all mTECs can present all peptides, but for lower degeneracies (*d* ⪅ *m*/*s*) it is best to divide the mTECs into *K* ≈ *m*/*s* classes, each of which can present approximately *s* peptides. The reason is as follows. For such degeneracies, the chance of finding two or more matching peptides in a random sample of *s* peptides from the whole pool becomes small. But if an mTEC has a limited repertoire of peptides then, if this set happens to contain one matching peptide, there is a much more significant chance that two copies of it will be presented. We can illustrate the general principle with the following toy problem.

Suppose there are just two distinct peptides, one of which is recognized (H), and one which is not (T), that an mTEC presents two peptides, and that a T-cell needs two hits to be negatively selected. If all cells can present both peptides then the probability of negative selection is 1/4: there are four possibilities for presentation HH, HT, TH, TT and only HH is negatively selected. Now suppose that in fact there are two types of mTEC, one of which can only present H and one which can only present T. Now the probability of negative selection is 1/2: there are only two possibilities for presentation: HH and TT.

Since high-degeneracy TCRs are easily removed, and the negative selection of low-degeneracy TCRs is enhanced by correlations in gene expression in mTECs, we anticipate that such correlation may be biologically advantageous. Of course, the overall efficiency of negative selection will depend on the degeneracy distribution of the incoming thymocytes: the system must find a balance between clearing out the majority of high-degeneracy thymocytes efficiently, and capturing the minority of low-degeneracy thymocytes before they exit.

The advent of single-cell sequencing means that gene-expression patterns in mTECs are now becoming available [2], and it may soon be possible to test some of our predictions. We view our work as a first step towards multi-scale models that can incorporate next-generation sequencing data and provide quantitative insights into the role of central tolerance in the immune system.

## Supplementary Material

Supporting information

## Data accessibility

No primary data were generated in this study. Simulation data and Mathematica notebooks which generate the figures are available at: https://doi.org/10.5287/bodleian:zryr1ZmmN.
