# Supplementary material for: Influence of correlated antigen presentation on T-cell negative selection in the thymus
Source: J R Soc Interface. 2018 Nov 7;15(148):20180311. doi: 10.1098/rsif.2018.0311 (PMC6283997; doi:10.1098/rsif.2018.0311)
Supplement: Supporting information [file rsif20180311supp1.pdf]

Supporting information: Influence of correlated antigen presentation on T cell negative selection in the thymus

Soumya Banerjee[1] and S. Jonathan Chapman[1]

1 Mathematical Institute, University of Oxford, Oxford, United Kingdom.

S1 Appendix. Probability of negative selection.

When $p = 2$, the probability of negative selection in a single interaction is

$$\begin{aligned} P_1 &\sim \sum_{r=0}^{m/K} \left(1-\frac{d}{m}\right)^{m/k-r} \left(\frac{d}{m}\right)^r \begin{pmatrix} m/K \\ r \end{pmatrix} \left(\left(1-\frac{rK}{m}\right)^s + \frac{sKr}{m}\left(1-\frac{rK}{m}\right)^{s-1} \right) \\ &\sim \sum_{r=0}^{m/K} \left(1-\frac{d}{m}\right)^{m/k-r} \left(\frac{d}{m}\right)^r \begin{pmatrix} m/K \\ r \end{pmatrix} \left(1+\frac{sKr}{m}\right) \mathrm{e}^{-rKs/m} \\ &\sim \left(1-\frac{d}{m}+\frac{d\mathrm{e}^{-sK/m}}{m}\right)^{m/K} \left(1+\frac{s}{1+(m/d-1)\mathrm{e}^{sK/m}}\right) \\ &\sim \exp\left(-\frac{d}{K}+\frac{d}{K}\mathrm{e}^{-sK/m}\right)\left(1+\frac{sd}{m}\mathrm{e}^{-sK/m}\right), \end{aligned}$$

giving Eq (8). For $p = 3$ we have instead

$$\begin{aligned} P_1 &\sim \sum_{r=0}^{m/K} \left(1-\frac{d}{m}\right)^{m/k-r} \left(\frac{d}{m}\right)^r \begin{pmatrix} m/K \\ r \end{pmatrix} \\ &\qquad \times \left(\left(1-\frac{rK}{m}\right)^s + \frac{sKr}{m}\left(1-\frac{rK}{m}\right)^{s-1} + \frac{s(s-1)K^2r^2}{2m^2}\left(1-\frac{rK}{m}\right)^{s-2} \right) \\ &\sim \sum_{r=0}^{m/K} \left(1-\frac{d}{m}\right)^{m/k-r} \left(\frac{d}{m}\right)^r \begin{pmatrix} m/K \\ r \end{pmatrix} \left(1+\frac{sKr}{m}+\frac{s(s-1)K^2r^2}{2m^2}\right) \mathrm{e}^{-rKs/m} \\ &\sim \left(1-\frac{d}{m}+\frac{d\mathrm{e}^{-sK/m}}{m}\right)^{m/K} \\ &\qquad \times \left(1+\frac{s}{1+(m/d-1)\mathrm{e}^{sK/m}} + \frac{s(s-1)}{2}\frac{((K/d-K/m)\mathrm{e}^{sK/m}+1)}{(1+(m/d-1)\mathrm{e}^{sK/m})^2}\right) \\ &\sim \exp\left(-\frac{d}{K}+\frac{d}{K}\mathrm{e}^{-sK/m}\right)\left(1+\frac{sd}{m}\mathrm{e}^{-sK/m}+\frac{s^2dK}{2m^2}\mathrm{e}^{-sK/m}\left(1+\frac{d}{K}\mathrm{e}^{-sK/m}\right)\right), \end{aligned}$$

which is Eq (9).

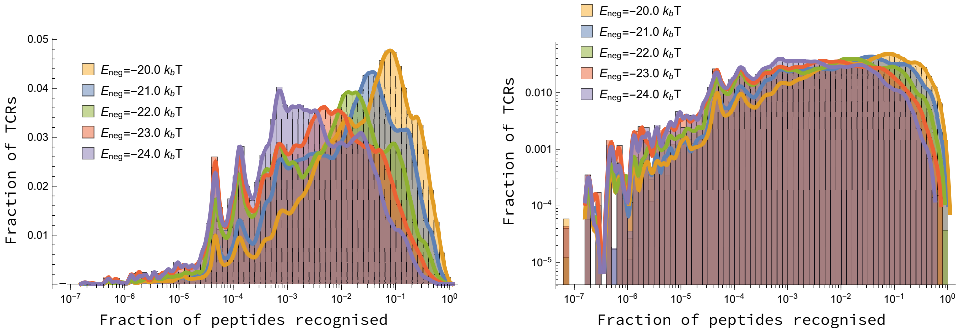

(a) (b)

S1 Fig. Distribution of the proportion of peptides recognised by each of 200,000 randomly generated TCR sequences shown on (a) log-linear and (b) log-log scales for a variety of values of E_{neg}, with $N = 5$. The proportion of TCRs not recognising any peptides was approximately 6%, 12%, 19%, 31% and 44% for $E_{\mathrm{neg}} = -20.0k_{\mathrm{b}}T$, $-21.0k_{\mathrm{b}}T$, $-22.0k_{\mathrm{b}}T$, $-23.0k_{\mathrm{b}}T$, and $-24.0k_{\mathrm{b}}T$, respectively.

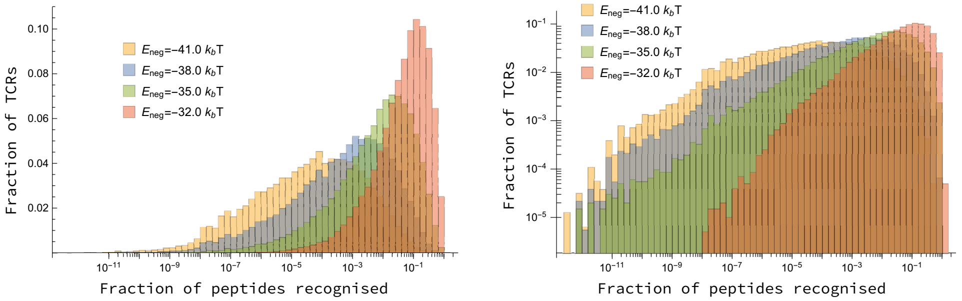

(a) (b)

S2 Fig. Distribution of the proportion of peptides recognised by each of 200,000 randomly generated TCR sequences shown on (a) log-linear and (b) log-log scales for a variety of values of E_{neg}, with $N = 9$. The proportion of TCRs not recognising any peptides was approximately 0%, 0.4%, 4.4%, and 21% for $E_{\mathrm{neg}} = -32.0k_{\mathrm{b}}T$, $-35.0k_{\mathrm{b}}T$, $-38.0k_{\mathrm{b}}T$, and $-41.0k_{\mathrm{b}}T$, respectively.

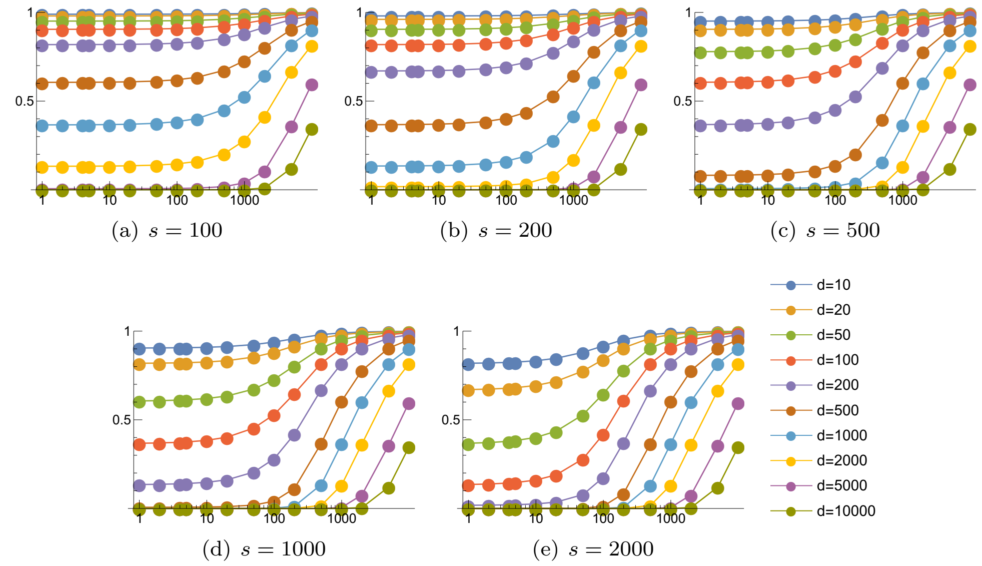

(a) $s = 100$ (b) $s = 200$ (c) $s = 500$

(d) $s = 1000$ (e) $s = 2000$

S3 Fig. Mean probability of escape in a single interaction, P_1, as a function of the number of mTEC classes, K, for various TCR degeneracies, d. Other model parameters are $m = 100000$, $p = 1$.

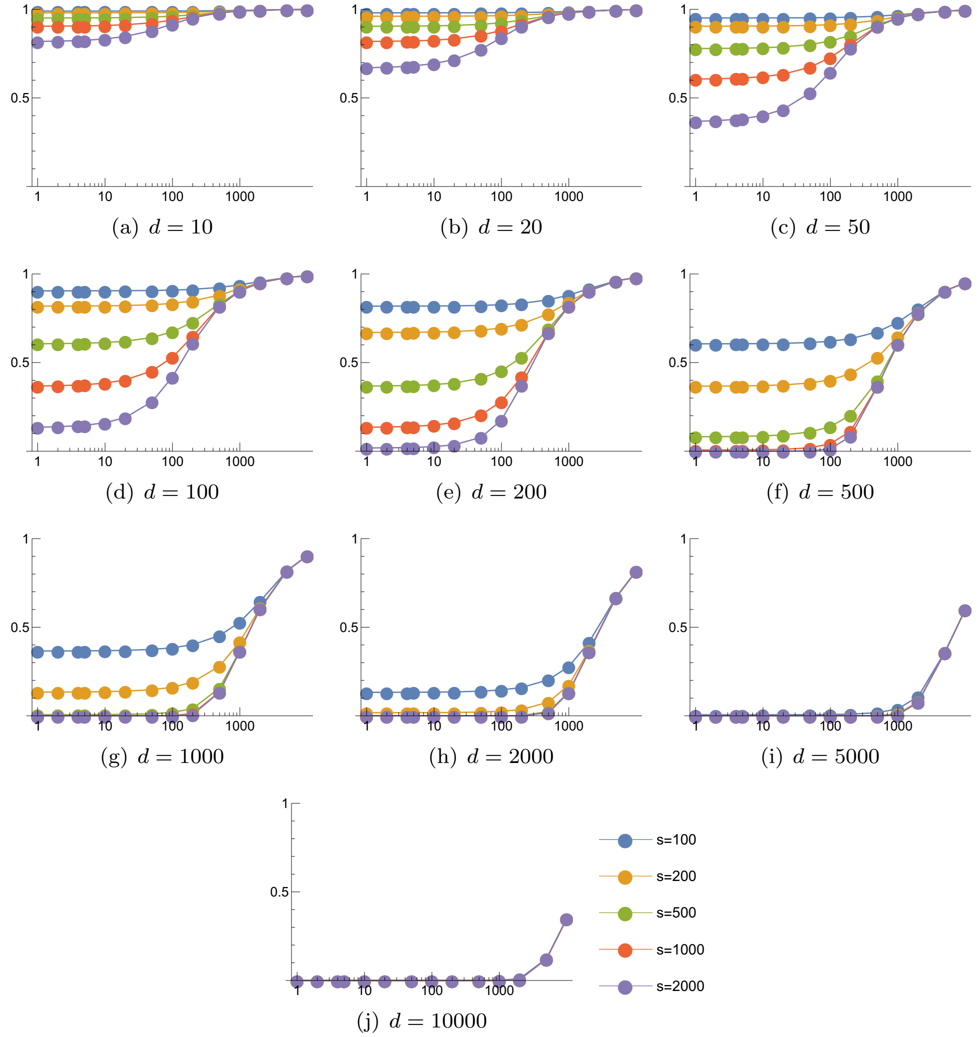

S4 Fig. Mean probability of escape in a single interaction, P_1, as a function of the number of mTEC classes, K, for various numbers of TCR-pMHC complexes in the immunological synapse, s. Other model parameters are $m = 100000$, $p = 1$.

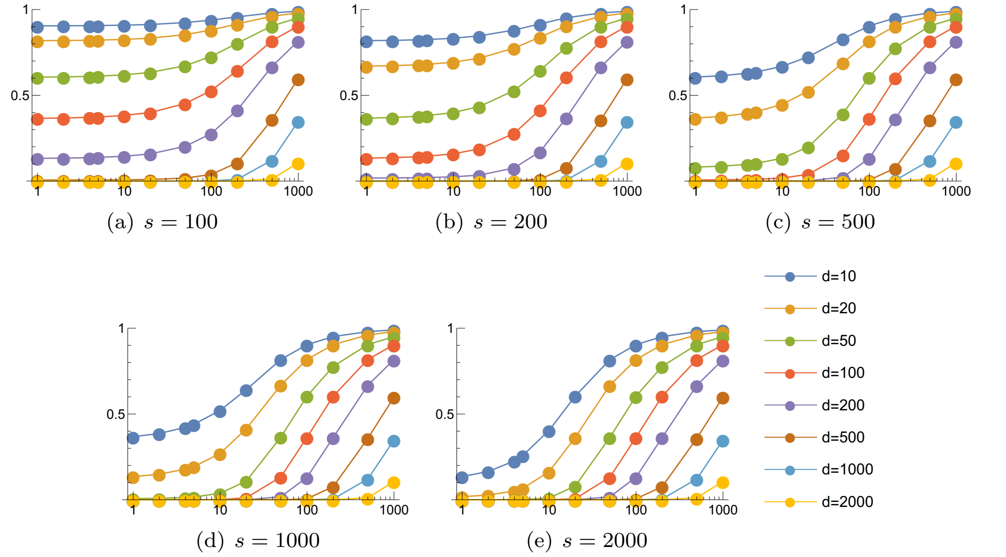

S5 Fig. Mean probability of escape in a single interaction, P_1, as a function of the number of mTEC classes, K, for various TCR degeneracies, d. Other model parameters are $m = 10000$, $p = 1$.

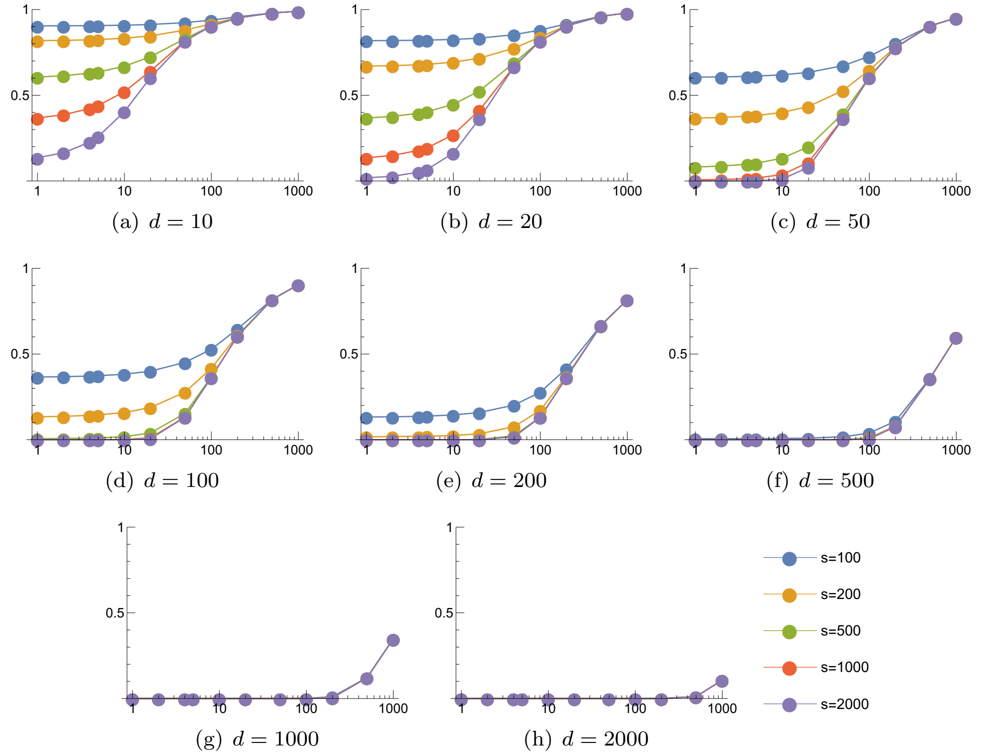

S6 Fig. Mean probability of escape in a single interaction, P_1, as a function of the number of mTEC classes, K, for various numbers of TCR-pMHC complexes in the immunological synapse, s. Other model parameters are $m = 10000$, $p = 1$.

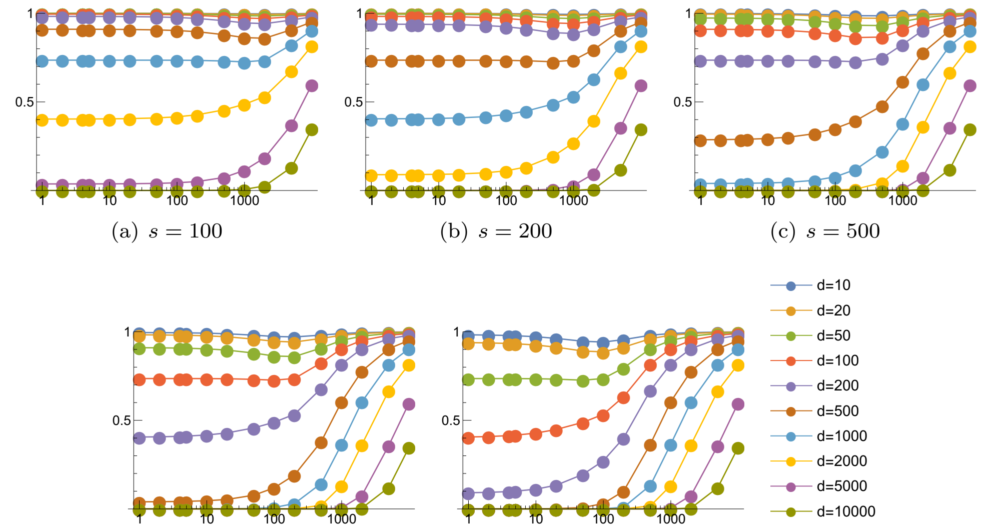

(a) $s = 100$ (b) $s = 200$ (c) $s = 500$

(d) $s = 1000$ (e) $s = 2000$

S7 Fig. Mean probability of escape in a single interaction, P_1, as a function of the number of mTEC classes, K, for various TCR degeneracies, d. Other model parameters are $m = 100000$, $p = 2$.

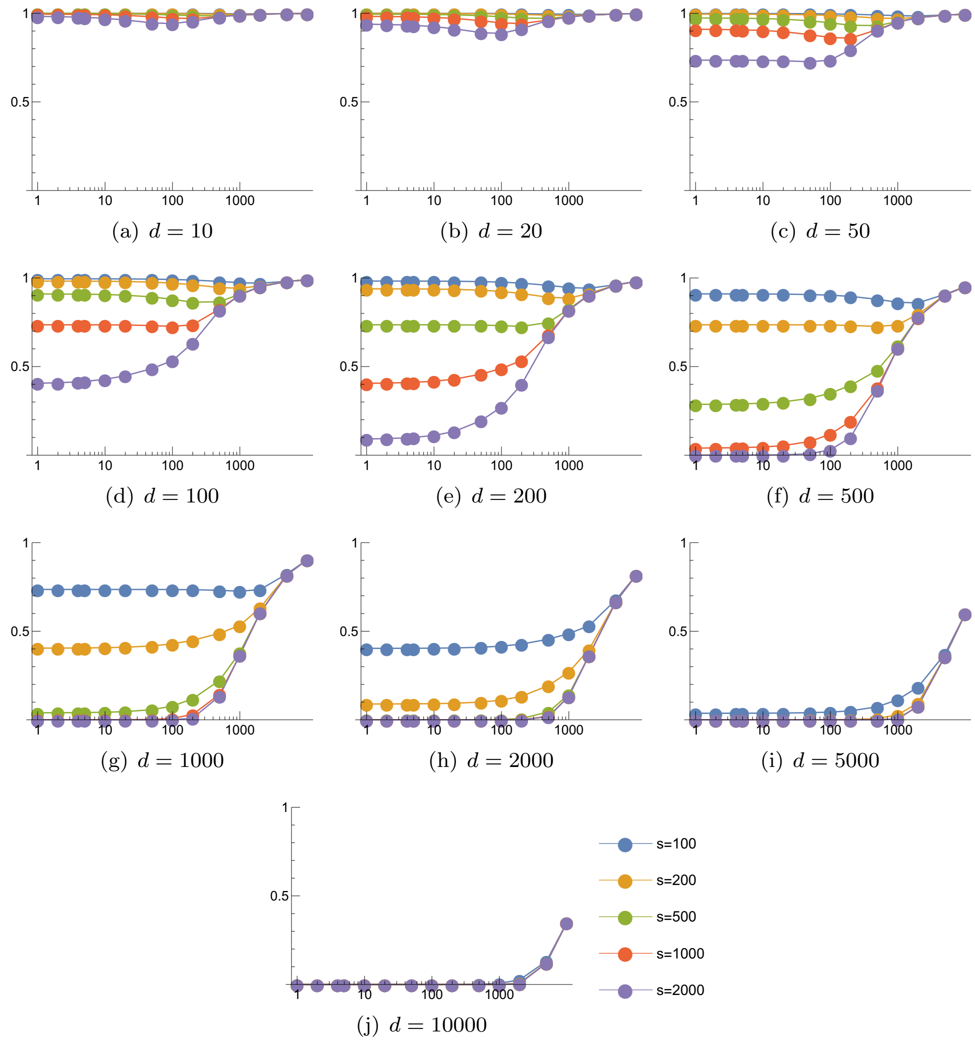

(a) $d = 10$ (b) $d = 20$ (c) $d = 50$

(d) $d = 100$ (e) $d = 200$ (f) $d = 500$

(g) $d = 1000$ (h) $d = 2000$ (i) $d = 5000$

(j) $d = 10000$

S8 Fig. Mean probability of escape in a single interaction, P_1, as a function of the number of mTEC classes, K, for various numbers of TCR-pMHC complexes in the immunological synapse, s. Other model parameters are $m = 100000$, $p = 2$.

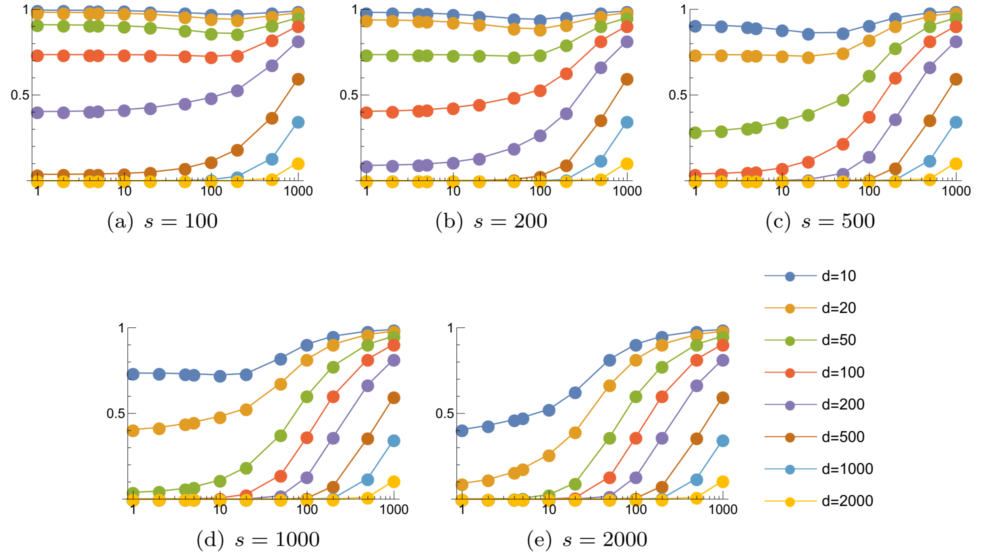

(a) $s = 100$ (b) $s = 200$ (c) $s = 500$

(d) $s = 1000$ (e) $s = 2000$

S9 Fig. Mean probability of escape in a single interaction, P_1, as a function of the number of mTEC classes, K, for various TCR degeneracies, d. Other model parameters are $m = 10000$, $p = 2$.

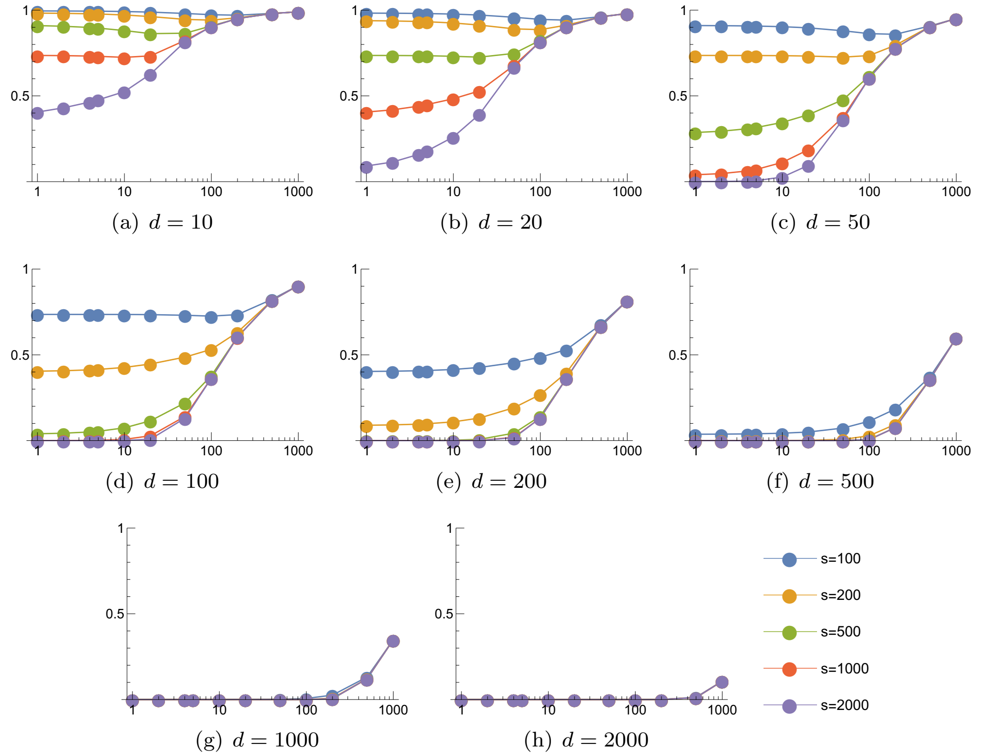

(a) $d = 10$ (b) $d = 20$ (c) $d = 50$

(d) $d = 100$ (e) $d = 200$ (f) $d = 500$

(g) $d = 1000$ (h) $d = 2000$

S10 Fig. Mean probability of escape in a single interaction, P_1, as a function of the number of mTEC classes, K, for various numbers of TCR-pMHC complexes in the immunological synapse, s. Other model parameters are $m = 10000$, $p = 2$.

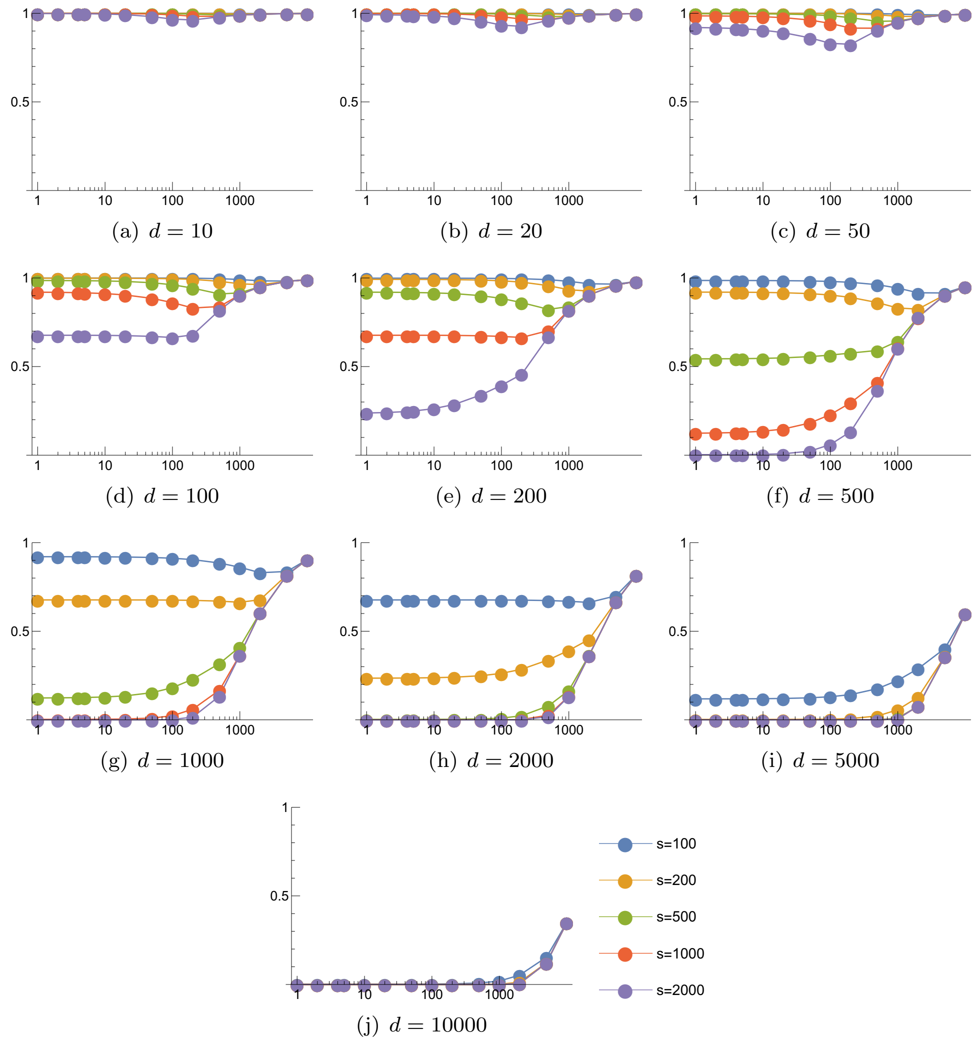

S11 Fig. Mean probability of escape in a single interaction, P_1, as a function of the number of mTEC classes, K, for various numbers of TCR-pMHC complexes in the immunological synapse, s. Other model parameters are $m = 100000$, $p = 3$.

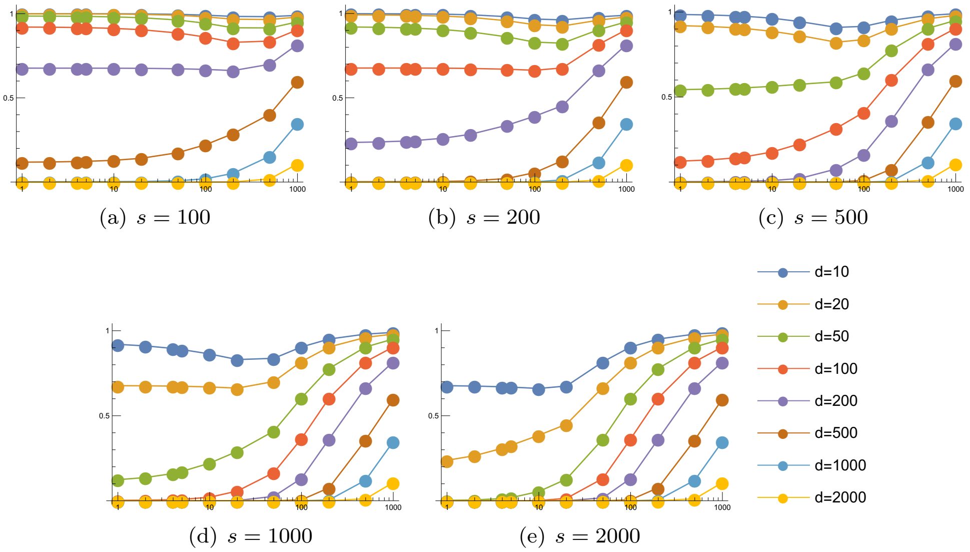

S12 Fig. Mean probability of escape in a single interaction, P_1, as a function of the number of mTEC classes, K, for various TCR degeneracies, d. Other model parameters are $m = 10000$, $p = 3$.

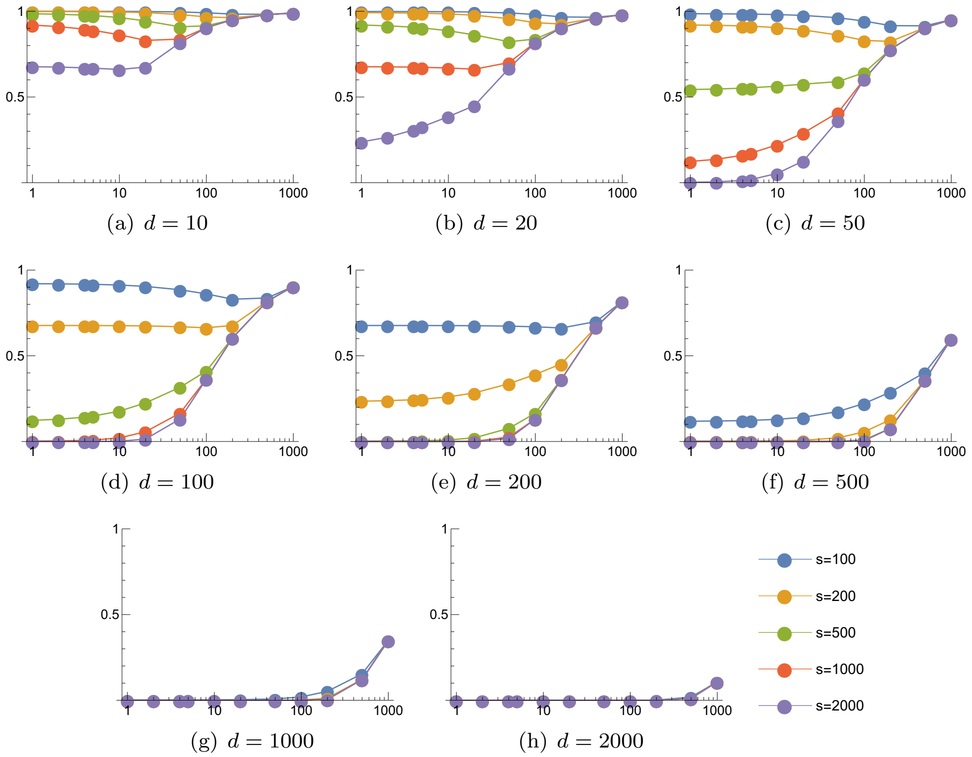

S13 Fig. Mean probability of escape in a single interaction, P_1, as a function of the number of mTEC classes, K, for various numbers of TCR-pMHC complexes in the immunological synapse, s. Other model parameters are $m = 10000$, $p = 3$.

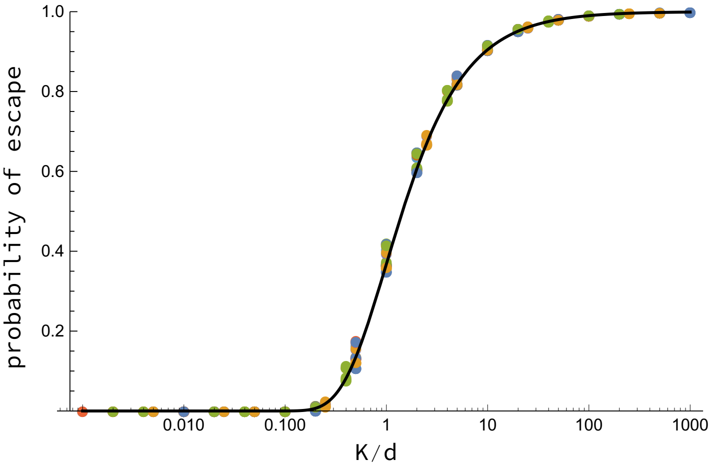

S14 Fig. The mean probability of escape as a function of K/d, illustrating the collapse of the data when $s > 2m/K$. The curve is given by Eq (6). The points are calculated using all those combinations of $s = 100$, 200, 500, 1000, 2000, $K = 1$, 2, 4, 5, 10, 20, 50, 100, 200, 500, 1000, $m = 10^4, 10^5$, and $d = 1$, 2, 5, 10, 20, 50, 100, 200, 500, 1000, 2000, 5000, 10000, 20000, 50000, 100000, which satisfy the constraint $s > 2m/K$ (and $d \leq m$). The plot shows data for $p = 1$. For $p = 2$ the requirement is more like $s > 3.5m/K$; for $p = 2$ the requirement is $s > 5m/K$.

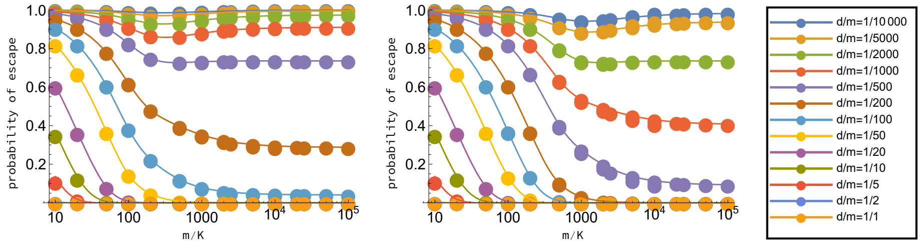

(a) $s = 500$ (b) $s = 2000$

S15 Fig. The mean probability of escape as a function of m/K. The curves are given by Eq (8). The points are calculated values, using both $m = 10^4$ and 10^5. For $s = 500$ these points lie on top of one another. For $s = 2000$ the difference between the two sets of points is just visible near $m/K = 10^4$. Here $p = 2$.
